# Outpatient mental health care during high incidence phases of the COVID-19 pandemic in Germany – changes in utilization, challenges and post-COVID care

**DOI:** 10.1007/s00406-024-01886-w

**Published:** 2024-09-01

**Authors:** Mandy Fehr, Sabine Köhler, Christa Roth-Sackenheim, Katharina Geschke, Oliver Tüscher, Kristina Adorjan, Klaus Lieb, Lars P. Hölzel, Hauke F. Wiegand

**Affiliations:** 1 Department of Psychiatry and Psychotherapy, University Medical Center of the Johannes Gutenberg-University Mainz, Untere Zahlbacher Straße 8, 55131 Mainz, Germany; 2 Berufsverband Deutscher Nervenärzte, Berlin, Germany; 3 Berufsverband Deutscher Psychiater, Berlin, Germany; 4 Department of Psychiatry and Psychotherapy, University of Bern, Bern, Switzerland; 5 Department of Psychiatry and Psychotherapy, University Hospital, Ludwig Maximilians University Munich, Munich, Germany; 6 Oberberg Parkklinik Wiesbaden Schlangenbad, Schlangenbad, Germany; 7 Department of Psychiatry, Psychotherapy and Psychosomatics, University Medicine Halle, Halle, Germany

**Keywords:** COVID-19, Mental health care, Outpatient care, Telemedicine, Post-COVID

## Abstract

**Background:**

As only a few studies have examined the impact of the COVID-19 pandemic on the mental health outpatient system so far, the aim of the *COVID Ψ Outpatient Survey* was to gain insight from outpatient providers in Germany regarding changes in utilization; associated problems and challenges; telemedicine services; interactions with inpatient and nursing home services; and experiences with post-COVID syndromes.

**Methods:**

Between July and September 2021, we invited 351 randomly selected outpatient mental health specialists to take part in the online survey via e-mail. Additionally, we extended an invitation to professional associations to encourage their members to participate. N = 105 physicians of most regions of Germany took part in the survey.

**Results:**

Survey participants reported changes in utilization during the high incidence phases (HIP) of the pandemic using pre-formulated categories: For the first HIP in spring 2020, 31% of the survey participants reported a decrease > 20% and 5% an increase > 20% of patient contacts. For the third HIP in spring 2021, 4% reported a decrease > 20% of contacts, while 30% an increase > 20%. Participants chose “patient’s fears of infection” and “providers protection measures” as reasons for decreases, and “pandemic related anxieties”, “economic stressors”, and “capacity reductions of the inpatient system” as reasons for increases of patient contact. Many providers introduced telemedicine services. A majority reported consultations for post-COVID syndromes already in spring 2021.

**Conclusions:**

The survey hinted at changes in utilization, multiple problems but as well good-practice-solutions in the mental health outpatient system during the COVID-19 pandemic.

**Supplementary Information:**

The online version contains supplementary material available at 10.1007/s00406-024-01886-w.

## Introduction

The COVID-19 pandemic was a major challenge for mental healthcare systems. During the first high incidence phase (HIP) in spring 2020, emergency and inpatient mental health services showed large reductions in utilization worldwide [1–15]. Notably, several studies observed a concurrent increase in acute and involuntary inpatient admissions [1, 3, 6, 8, 16]. For the German inpatient mental health care system, similar patterns were found: For the first HIP in spring 2020, both survey and routine data studies found decreases in hospital admissions [17–19], but a relative increase in involuntary and urgent admissions [20, 21]. In a survey of department heads of German psychiatric inpatient institutions, similar reductions to 80% of pre-pandemic utilization were reported for the second HIP in winter 2020/2021. Problems resulting from this reduced inpatient utilization were a lack of integration of patients into their living environment, disease exacerbations, loss of contact, suicide attempts, and insufficient outpatient treatment alternatives [20].

Studies on mental health outpatient services, nearly all from the UK, consistently showed significant reductions in referrals to primary and secondary mental health services and in incidence diagnoses of mental disorders during the first HIP [22–28], but some studies showed increases in referrals after this period [22, 24, 25]. One routine data report from Germany showed a decrease in the number of individual and group psychotherapy cases and the number of psychiatric treatment cases in spring 2020 [29]. Not many studies from other regions of Europe exist and no study examined so far which further challenges occurred to mental health outpatient providers in Europe during the COVID-19 pandemic.

Changes in utilization of the outpatient mental healthcare system might have been due to changes in inpatient treatment capacities and utilization, but other factors like fears of infection might have kept patients from on-site consultations, non-essential contacts might have been reduced as an infection protection measure [20], and due to the enabling of telemedicine consultations. Furthermore, several reports suggested an increasing burden of mental disorders in the general population over the course of the COVID-19 pandemic due to side effects of lockdown measures and economic hardships [30–36]. Post-COVID syndromes often comprise psychopathological symptoms that might have led to consultations of outpatient mental health specialists [37–42]. In addition, some populations of people with pre-existing mental disorders seemed to be especially vulnerable for worsening of their mental health [43, 44].

To learn more about utilization and problems in the mental health outpatient system in the different phases of the COVID-19 pandemic, we initiated the *COVID Ψ Outpatient Survey* that aimed at surveying outpatient mental health specialists. We chose a survey format to complement routine data analyses with background information and to learn about the challenges experienced by the outpatient specialists. The survey aimed at examining utilization during and in between the first three HIPs in Germany in spring 2020, winter 2020/2021 and spring 2021; problems, challenges, and contributing factors associated with changes in utilization; experiences with telemedicine services; problems in interactions with inpatient mental health facilities and changes in psychiatric care in the nursing homes and assisted living sectors; and experiences with demand from patients suffering from post-COVID syndromes.

## Methods

### Study design and participants

To find contact details of outpatient specialists for psychiatry and psychotherapy (“Facharzt für Psychiatrie und Psychotherapie”), psychiatry and neurology (“Facharzt für Nervenheilkunde”, “Nervenarzt”), and psychosomatic medicine and psychotherapy (“Facharzt für psychosomatische Medizin und Psychotherapie”), we used the websites of the Association of Statutory Health Insurance Physicians (Kassenärztliche Vereinigung) of the 16 federal states of Germany, between July and September 2021. From these, we randomly selected n = 351 and invited them by email to participate in an online survey. A prerequisite for the selection was the existence of a valid email address. As the survey aimed at descriptive results, the only selection criterion was the attempt to invite participants from all postal code regions in Germany. Additionally, the BVDP (Bundesverband Deutscher Psychiater) and BVDN (Bundesverband Deutscher Nervenärzte), professional associations of mental health specialist physicians in Germany, invited their members by email to take part in the survey. To ensure representativity, mental health specialists from most postal code regions in Germany were included. A detailed breakdown of the federal states in which the participants work is provided in Online Resource 1. The questionnaire was anonymous. The survey was realized in LymeSurvey^®^ on the data-protected servers of the University Medical Center Mainz. An English translation of the original German survey questions is provided in Online Resource 2.

### Indicators and outcomes

The survey questions were developed based on the results of a first survey which was conducted right after the start of the pandemic [17], a screening of the international literature, and structured discussions between the authors, who have different backgrounds in psychiatric outpatient (C. R. S., S. K.), psychiatric inpatient (O. T., K. L., K. A., H. F. W.), private psychiatric inpatient with a focus on psychotherapy (L. P. H.), and geriatric psychiatry (K. G.) services. The literature was screened using a systematic search strategy that was further developed into [45]; this updated literature review will be published separately. The survey covered the topics changes in service utilization, problems by changes in utilization, use of telemedicine, referrals to inpatient institutions, medical attendance in nursing homes and assisted living institutions, and post-COVID-syndromes in outpatient psychiatry. The questions were designed with pre-formulated answers and an option of free-text answers. This design was chosen to identify additional problems and outcomes that were not pre-formulated in this questionnaire.

### Analysis

Microsoft Excel 16.79.2 was used for graph generation and statistical analyses. The results (percentages) of each of the pre-formulated response refer to the total number of participants that responded to it, unless otherwise indicated. Free-text responses were examined by qualitative content analysis following Mayring [46]. The answers were discussed together and grouped into main groups according to their content and context. The answers were then further categorized independently by two authors (M. F. and L. P. H.). Conflicting categorizations were discussed and jointly resolved by three authors (M. F., L. P. H. and H. F. W.).

## Results

### Profile of the participating mental health specialists

Out of the 351 personally contacted physicians (plus those who followed the invitation in a general group email to the members of BVDP and BVDN), a total of n = 105 specialists for psychiatry and psychotherapy (“Facharzt für Psychiatrie und Psychotherapie”), psychiatry and neurology (“Facharzt für Nervenheilkunde”, “Nervenarzt”), and psychosomatic medicine and psychotherapy (“Facharzt für psychosomatische Medizin und Psychotherapie”) took part in the survey. Table 1 shows their specialization and Online Resource 1 a breakdown of the federal states in which the participants work.

**Table 1:** Characteristics of the survey’s participants (The sum of the percentages can be more than 100%.)

Health insurance accreditation	% of the participants n = 105
Psychiatry	70.5%
Psychosomatic medicine	4.8%
Psychiatry and neurology (“Nervenheilkunde“)	33.3%
Neurology	29.5%
Psychotherapy	54.3%

Type of psychotherapy accreditation of participants with health insurance accreditation for psychotherapy	% of the participants n = 57
Cognitive behavioral therapy	43.9%
Brief psychodynamic therapy	64.9%
Psychoanalysis	3.5%
Systemic therapy	5.3%
Other forms of psychotherapy	5.3%

Offer of individual and/or group therapy	% oft the participants n = 57
Individual therapy	98.2%
Group therapy	15.8%

### Service utilization

**Fig. 1:**
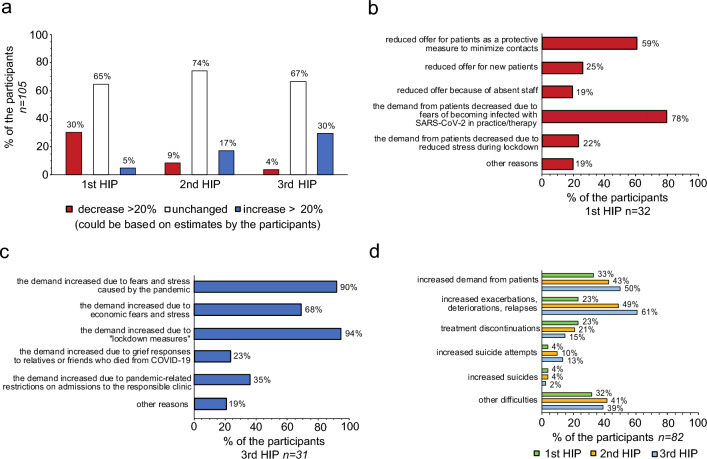
Changes in Utilization: **a** changes in the number of contacts compared to 2019 (the selected category could be based either on evaluations of routine data from individual participants records or on their estimates) **b** reasons for a decrease in the amount of contacts c reasons for an increase in the amount of contacts d difficulties and complications due to reduced inpatient and outpatient offerings during the first three high incidence phases (HIP); 1st HIP: spring 2020, 2nd HIP: winter 2020/2021, 3rd HIP spring 2021

First, we asked if the number of contacts to patients changed during the three HIPs of the COVID-19 pandemic compared to 2019. The participants could select from the pre-formulated categories “increase > 20%”, “no change”, and “decrease > 20%”, the selected category could have been based on evaluations of routine data from individual participants records or on their estimates. The majority indicated an unchanged number of contacts throughout all three high incidence phases (HIP): 1st HIP 64.8% (n = 68), 2nd HIP 74.3% (n = 78), 3rd HIP 66.7% (n = 70). However, during the first HIP, 30.5% (n = 32) reported a relevant decrease > 20%, whereas during the third HIP, 29.5% (n = 31) reported a relevant increase > 20%. See Fig. 1a for the detailed results.

Subsequently, we evaluated possible causes of the decrease in contacts for each HIP separately. Participants who indicated a decrease in contacts in a HIP could select pre-formulated responses or enter additional free-text: “Patient demand decreased due to fears of becoming infected with SARS-CoV-2 in practice/therapy”, “Patient demand was reduced due to reduced stress during lockdown”, “The offer was reduced for patients as a protective measure to minimize contacts”, “The offer was reduced for new patients”, “The offer was reduced because staff was absent”. During the first HIP, 78.1% (n = 25) attributed the decrease to a reduced demand from patients due to fears of becoming infected with SARS-CoV-2 in practice/therapy, and 59.4% (n = 19) to a reduced offer as a protective measure to minimize contacts. See Fig. 1b for the detailed results.

Next, we asked for reasons for the increasing number of contacts. Participants who indicated increases in contacts could select again pre-formulated responses or enter additional free-text: “Demand increased due to pandemic-related fears and stress”, “Demand increased because of economic concerns and stress”, “Demand increased as a consequence of lockdown measures” and “Demand increased due to pandemic-related restrictions on admissions by the local inpatient departments/clinics”. Most frequently selected responses during the third HIP were an increase in demand because of lockdown measures (93.5%; n = 29), due to pandemic-related fears and stress (90.3%; n = 28), and due to economic concerns and stress (67.7%; n = 21). See Fig. 1c for the detailed results.

Of all participants (n = 105), 41.9% (n = 44) reported a change in service utilization among certain patient groups (both previously known and previously unknown) during or after the first HIP, 29.5% (n = 31) during or after the second HIP, and 36.2% (n = 38) during or after the third HIP. In free-text answers, less contacts were mentioned, especially for the first and second HIP, for patients with addiction disorders, depression and anxiety disorders, previously known patients, those of higher age and those living in retirement homes. More contacts were reported, especially for the second and third HIP, for patients with depressive and anxiety disorders, those who were affected by the closure of psychosocial and self-help institutions and a reduced offer of inpatient mental healthcare intuitions, previously unknown patients, and patients of younger age. We identified certain groups with a higher demand, e.g. (single) parents due to the closure of childcare institutions, socially isolated persons like students because of a lack of real-life contacts, and those with post-COVID-syndromes. For a detailed description, see Online Resource 3.

### Problems related to changes in utilization

We additionally asked about perceived complications and difficulties for the patients due to the pandemic-related adjustments in the outpatient and inpatient mental health care system. Overall, 78.1% (n = 82) of the participants reported difficulties. Participants who indicated difficulties in a HIP could select the following pre-formulated responses or enter additional free-text: “increased demand from patients”, “increased exacerbations”, “deteriorations and relapses”, “treatment discontinuations and loss of contact” and “increases in suicide attempts and suicides”. Most frequently reported difficulties were an increased demand from patients (1st HIP 32.9%, n = 27; 2nd HIP 42.7%, n = 35; 3rd HIP 50%, n = 41) and increased exacerbations (1st HIP 23.2%, n = 19; 2nd HIP 48.8%, n = 40; 3rd HIP 61%, n = 50). See Fig. 1d for the results. In free-text answers, the outpatient specialists marked that there were difficulties to transfer patients to inpatient and day-clinic institutions, an increase in the already before existing difficulties in finding psychotherapy offerings, closures of psychosocial and self-help offers but as well a deterioration of the doctor-patient relationship due to the hygiene measures. For a detailed description, see Online Resource 3.

### Telemedicine

**Fig. 2:**
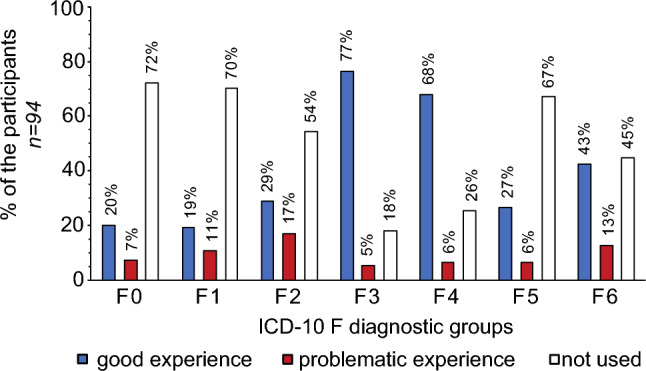
Experiences with telemedicine for specific ICD-10 F groups F0: organic, including symptomatic, mental disorders, F1: addictive disorders, F2: schizophrenia, schizotypal and delusional disorders, F3: affective disorders, F4: neurotic, stress-related, and somatoform disorders, F5: eating disorders, F6: personality disorders

Regarding telemedicine use before, during and (planned) after the pandemic, out of the n = 99 participants, telephone consultation services were used by 37.1% (n = 37) before the pandemic, 55.6% (n = 55) newly introduced them during the pandemic, 27.3% (n = 27) planned to further use them after the pandemic, and 9.1% (n = 9) did not use them at all. Video consultation services were used by 3% (n = 3) before the pandemic, 49.5% (n = 49) newly introduced them during the pandemic, 22.2% (n = 22) planned to use them further after the pandemic, and 47.5% (n = 47) did not used them at all. Self-help apps for patients were used by 5.1% (n = 5) before the pandemic, 14.1% (n = 14) newly introduced them during the pandemic, 12.1% (n = 12) indicated a further planned use after the pandemic, and 78.8% (n = 78) did not use them at all. Results on the use and experiences of telemedicine services for specific diagnostic groups (n = 94 participants) are shown in Fig. 2.

### Referrals to inpatient institutions

Asked how transferals from the outpatient specialists to mental health inpatient institutions changed during the pandemic, out of the n = 96 participants, a reduction in transferals was reported by 61.5% (n = 59) for the first HIP, by 54.2% (n = 52) for the second HIP, and by 38.5% (n = 37) for the third HIP. No participants reported increases in transferals for the first HIP, but 2.1% (n = 2) and 8.3% (n = 8) for the second and third HIP.

We additionally asked the participants who indicated a reduction in transferals to hospitals about possible reasons for these changes with pre-formulated responses: 87.1% (n = 54) of the participants (n = 62) reported reduced offer by the hospitals as a reason for fewer admissions, 67.7% (n = 42) less demand from patients because of fear of getting infected with SARS-CoV-2 in the hospital, and 66.1% (n = 41) less demand from patients due to restrictions during inpatient treatment (e.g., because of hygiene measures). 24.2% (n = 15) reported having been reluctant themselves to initiate transferals due to restrictions of inpatient treatment (e.g., due to hygiene measures), 12.9% (n = 8) reported having been reluctant themselves out of concern for patient infections in the hospital, and 9.7% (n = 6) reported less demand due to a lower burden of disease.

### Medical attendance in nursing homes and assisted living institutions

Out of the n = 96 participants, 66.7% (n = 64) reported that they provided mental health care for old people’s homes, nursing homes, or complementary facilities such as assisted living for people with mental illness. Asked how their provision of mental health care for those institutions had changed during the pandemic, 45.3% (n = 29) reported no change, 50% (n = 32) fewer medical visits, and 4.7% (n = 3) more medical visits. When asked why there had been fewer medical visits, the participants, could select out of pre-formulated responses. Out of the participants who previously reported fewer medical visits (n = 32), 75% (n = 24) of the participants reported that the facilities did not want visits as a COVID-19 protective measure, 59.4% (n = 19) that they had visited as few facilities as possible as a self-initiated COVID-19 protective measure, and 9.4% (n = 3) each stated less demand from patients due to COVID-19 outbreaks, less demand because patients were in inpatient treatment due to COVID-19, and less demand from patients because they died from COVID-19. 6.3% (n = 2) reported less demand from patients due to fear of SARS-CoV-2 infection, and 3.1% (n = 1) less demand due to a decrease in mental illnesses or exacerbations.

Furthermore, we evaluated the need for inpatient admission of inhabitants of nursing homes and assisted living facilities. Out of the participants who provided mental health care within these facilities (n = 62), 20.3% (n = 13) reported a change in the need for inpatient admission for psychiatric patients, 71.9% (n = 46) stated no difference in the need for inpatient admission, and 7.8% (n = 5) did not know if there was a change.

### Post-COVID syndromes

**Fig. 3:**
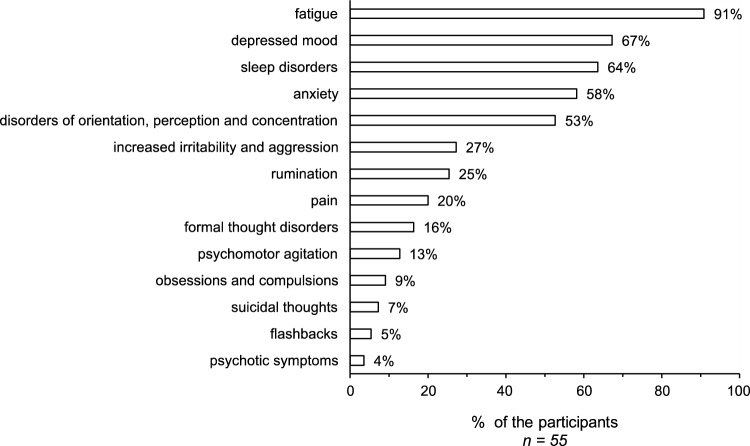
Psychopathology of post-COVID syndromes in routine care

55% (n = 55) of the responding participants (n = 100) affirmed that they had already treated patients whose mental illness could be classified as a result of a confirmed SARS-CoV-2-infection (post-COVID syndrome). The most observed psychopathology was fatigue (91%, n = 49), depressed mood (67%, n = 36), and sleep disorders (64%, n = 35). For further results, see Fig. 3. Asked about the treatment of these post-COVID syndromes, 90.9% (n = 50) stated that they offered supportive conversations, 69.1% (n = 38) used both antidepressants as well as psychotherapeutic interventions, 58.2% (n = 32) reported transferals to rehabilitation treatments, 40% (n = 22) transferals to complementary treatments (e.g., occupational or physical therapy), 16.4% (n = 9) used sedative medication, and 5.5% (n = 3) high potency antipsychotics. 7.3% (n = 4) reported that they filed out retirement applications for their patients.

## Discussion

The COVID Ψ outpatient Survey identified several important mental healthcare challenges during the first three HIPs of the COVID-19 pandemic in Germany, drawn from the answers of a regionally diverse sample of psychiatric specialists.

### Changes in utilization

Overall, the results suggested a stable number of contacts and therefore continuity of care for psychiatric outpatient consultations. However, the second most selected answer was a decrease of contacts during the initial HIP. In both standard and free-text responses, patients’ fear of infection and the reduced services offered by outpatient providers were given as reasons for decreases. During the second and third HIP, some specialists reported an increase in contacts. Reasons indicated most frequently by the participants were worries because of the pandemic itself, stress related to economic worries and related to lockdown measures such as social isolation. The free-text answers identified certain groups with a higher demand, e.g. (single) parents, students, and people with pre-existing mental disorders. The outpatient specialists also reported being more reluctant with transferals to inpatient institutions, mainly due to the reduced offer of these institutions and patients’ fears of infection during inpatient treatment. These results were mainly in line with the evolution of mental health care utilization in other regions in Europe [47]. Going along with these changes in utilization, an increase of problems was perceived due to reduced services over the course of the pandemic: Whereas for the first HIP only a quarter of the participants reported exacerbations and 3% suicide attempts, these statements were endorsed by 60% and 13%, respectively, for the third HIP. Interestingly, the number of participants reporting “cases of treatment discontinuation” decreased over the course of the pandemic. Apparently, the system adapted to the circumstances.

Overall, the reported problems like treatment discontinuations and suicide attempts, increases in demand due to reduced inpatient capacities but at the same time no increase in outpatient capacities, were in line with the results of a survey of inpatient institutions [20].

The reported delayed increase in demand matches the development of suicide rates in Germany, which were still at the low level of previous years in 2020 and 2021 but rose by 10% in 2022 [48]. These complications highlight the lack of regional oversight, coordination, and integration between inpatient and outpatient system as one of the central structural problems of (mental) health care in Germany that was aggravated by the crisis of the COVID-19 pandemic.

### Telemedicine

A majority of outpatient providers flexibly introduced or expanded telemedicine offerings and reported mostly good experiences, for e.g. affective and anxiety disorders. However, experiences seemed to have been mixed for patients with e.g. schizophrenia, schizotypal and delusional disorders, and organic (including symptomatic) mental disorders. Interestingly, despite the positive experiences, many providers do not plan to continue their telemedicine offerings. Reasons were not asked in this survey and can only be assumed. A user-friendly infrastructure and low bureaucracy refunding seem to be central. Furthermore, on-site (and home-treatment) offers for patient groups with limited financial and technical resources remain important [49–55].

### Reduced visits in nursing homes and assisted living institutions

Reports suggested that inhabitants of nursing homes were one of the neglected groups of the pandemic, with high COVID-19-associated mortality and social isolation rates [56–58]. The indicated reductions in on-site visits of inhabitants of nursing homes and assisted living institutions hint at a reduced quality of psychiatric care. In future crises, the necessities of infection control and psychiatric care of these vulnerable populations should be better balanced.

### Post-COVID syndromes in psychiatric care

The German S2k guideline on SARS-Cov-2, COVID-19 and (early) rehabilitation adopts the WHO’s post-COVID definition: The “condition occurs in individuals with a history of probable or confirmed SARS-CoV-2 infection, usually 3 months from the onset of COVID-19 with symptoms that last for at least 2 months and cannot be explained by an alternative diagnosis. Symptoms may be new onset, following initial recovery from an acute COVID-19 episode, or persist from the initial illness” [59]. However, at the time of the survey in summer 2021, definitions were still ambiguous, and therefore the survey asked just whether the mental health specialists had consultations for assumed post-COVID syndromes and for the observed broad psychopathological categories. More than 55% of the participants reported consultations for post-COVID syndromes. The most often reported symptoms like fatigue, depressed mood, or sleep deficits are in line with what is known about post-viral infection syndromes. The most used interventions were supportive counseling, antidepressant medications, psychotherapeutic interventions, and transferals to rehabilitation and complimentary treatments. These treatments seem largely adequate and are also recommended in the current S2k guideline [59]. As mental health specialists seem to be an important resource for patients with these syndromes, they should be included in the further development and evaluation of care standards for these syndromes.

### Limitations

This survey has several limitations. As the survey aimed at descriptive results, except for the attempt to gain a certain regional representativity no further attempts to balance the sample were made. The relatively moderate response rate (n = 105 of 351 contacted personally via mail and more via a newsletter) opens the possibility of response bias (e.g., only those with less demand took part in the survey). However, regional differences in pandemic response measures should be represented in the survey. The use of fixed values (increase/decrease > 20%) limits the interpretation of changes in service utilization: for example, regional changes might have been more subtle or much higher. Validity of these utilization results is limited as we cannot ensure that responses were based on evaluations of actual data and not only participants’ estimates. The questions and pre-formulated answers were based on the literature available at the time and the authors’ perspectives, which might have led to bias as well. However, the free-text answers gave participants opportunities to name further problems, thereby mitigating this bias possibility. Free-text answers were categorized using the widely used Mayring method. This method can lead to a loss of focus on the entirety of the text or individual case reports. In addition, the method is not suitable for developing a theory (like e.g., the grounded theory method). Since we neither had to categorize complex texts nor had the intention to develop a theory, Mayring's method seems appropriate for this analysis. Furthermore, this survey included questions about thoughts and actions of patients answered by their mental health specialists. This indirect reporting could lead to selective perception and confirmation bias (e.g., participants might only mention the information that supports their own assumption) as well as proxy bias (e.g., attributing own experiences onto the patients’ perspectives). Nevertheless, the survey format is providing important background information that is complementing more valid quantitative data sources like routine data.

## Conclusions

Taken together, the COVID Ψ Outpatient Survey results show that outpatient providers managed to maintain continuity of care with trends towards a decrease of utilization in the pandemic’s initial phase but later increases in demand. They show a difficult interplay between inpatient, outpatient, and assisted living sectors and problems for specific patient groups as well as providers resulting from these changes. They also show positive trends like the fast and flexible system-wide introduction of telemedicine offerings by outpatient specialists. Already during the first 1.5 years of the COVID-19 pandemic, outpatient psychiatric specialists saw a strong demand from patients suffering from post-COVID syndromes. Therefore, psychiatry and psychosomatic medicine should play a central role in the further development and evaluation of treatment standards for these syndromes. The outpatient provider’s views expressed in the survey are an important perspective of the systems burdens in times of crisis. The most important task for better managing future crises seems to establish structures for regional surveillance and coordination so that changes can be detected, and coordinated regional solutions developed in a more timely manner.

## Supplementary Information

Below is the link to the electronic supplementary material.

Supplementary file 1 (PDF 115 kb)

Supplementary file 2 (PDF 419 kb)

Supplementary file 3 (PDF 168 kb)

## Data Availability

The raw data supporting the conclusions of this article can be found here: https://github.com/haukefelixwiegand/COVID-Psy-outpatient-survey.git
