# Supplementary material for: Outpatient mental health care during high incidence phases of the COVID-19 pandemic in Germany – changes in utilization, challenges and post-COVID care
Source: Eur Arch Psychiatry Clin Neurosci. 2024 Sep 1;274(8):2025–35. doi: 10.1007/s00406-024-01886-w (PMC11579151; doi:10.1007/s00406-024-01886-w)
Supplement: Supplementary file 1 — (PDF 115 kb) [file 406_2024_1886_MOESM1_ESM.pdf]

Supplementary Information
Online Resource 1

Outpatient Mental Health Care During the First Three High Incidence Phases of the COVID-19 Pandemic in Germany - Results from the COVID Ψ Outpatient Survey

Mandy Fehr[1], Sabine Köhler[2,3], Christa Roth-Sackenheim[3,2], Katharina Geschke[1], Oliver Tüscher[1], Kristina Adorjan[4], Klaus Lieb[1], Lars P. Hölzel[1,5] and Hauke F. Wiegand[1]

[1]Department of Psychiatry and Psychotherapy, University Medical Center of the Johannes Gutenberg-University Mainz, Germany

[2]Berufsverband Deutscher Nervenärzte

[3]Berufsverband Deutscher Psychiater

[4] Department of Psychiatry and Psychotherapy, University Hospital, Ludwig Maximilians University Munich, Munich, Germany

[5]Oberberg Parkklinik Wiesbaden Schlangenbad, Schlangenbad, Germany

Keywords: COVID-19; pandemic; mental health care; outpatient care; psychiatry; telemedicine; Long COVID; COVID Vaccination

Corresponding author:

Hauke Felix Wiegand
Department of Psychiatry and Psychotherapy,
University Medical Center of the Johannes Gutenberg-University Mainz
Untere Zahlbacher Straße 8
55131 Mainz
Germany
+49 (0) 6131 177511
Haukefelix.wiegand@unimedizin-mainz.de

COVID-Ψ outpatient care

Guiding principles for the survey:

1. Identification of the current state of care provided by outpatient psychiatrists, neurologists, and psychotherapists during the pandemic
2. Identification of problem areas and good-practice solutions of care in the pandemic

a. Generally:

1. For which medical disciplines does your practice have a health insurance license ? (multiple answers possible)

- o Psychiatry
- o Psychosomatic medicine
- o Psychiatry and neurology
- o Neurology
- o Psychotherapy

2. If psychotherapy was indicated: What type of psychotherapy do you offer ? (multiple answers possible)

- o Cognitive behavioral therapy
- o Brief psychodynamic psychotherapy
- o Psychoanalysis
- o Systemic therapy
- o Other school of psychotherapy

3. If psychotherapy was indicated: Do you provide individual therapy and/or group therapy ? (if both apply, check both)

- o Individual therapy
- o Group therapy

4. Please provide the first two digits of your practice's zip code. (This data will only be used for correlation with maximum regional COVID-19 incidence, no analyses will be performed on individual practices). Not mandatory

Free text

b. Offer and service utilization:

5. If you use 2019 as a comparison, did the number of contacts in your practice change during the 1st, 2nd and 3rd High Incidence Phase (HIP)? (Both due to changes in the services you offer, e.g. due to hygiene-related restrictions, and due to changes in the use of services by patients)

	Relevant increase (e.g. > 20 %)	Relevant decrease (e.g. > 20 %)	Essentially unchanged
1st HIP March/April 2020			
2nd HIP November 2020 to February 2021			
3rd HIP March to Mai 2021			

6. If "decreased" is selected at 5. for one of the HIP: What do you attribute the described decrease to? (multiple selection possible, no mandatory selection in each line)

	1st HIP March/April 2020	2nd HIP November 2020 to February 2021	3rd HIP March to Mai 2021
The offer has been reduced for all patients as a protective measure to minimize contacts			
The offer was reduced for new patients (no acceptance of new patients)			
The offer was reduced because of absent staff			
Demand from patients decreased due to reduced stress during lockdown			
Demand from patients decreased due to fears of becoming infected with SARS-CoV-2 in practice/therapy			
Supply or demand declined for other reasons	Free text	Free text	Free text

7. If "increased" is selected for 5. in one of the HIP: What do you attribute the described increase to? (multiple selection possible, no mandatory selection in each line)

	1st HIP March/April 2020	2nd HIP November 2020 to February 2021	3rd HIP March to Mai 2021
The demand from some patient groups increased due to fears and stress caused by the pandemic			
The demand from some patient groups increased due to economic fears and stress			
The demand from some patient groups increased due to "lockdown measures" (e.g. loneliness, lack of social contacts, less activating, structuring outpatient offers, closure of day care centers, low-threshold offers, reduction of care)			
Demand from some patient groups increased due to grief responses to relatives or friends who died from COVID-19			
The demand from some patient groups increased due to pandemic-related restrictions on admissions to the responsible clinic			
Supply or demand increased for other reasons	Free text	Free text	Free text

8. Did certain groups of patients (both new and previously known) use the services of your practice in or after the 1st HIP in a significantly different way (e.g. significantly more or fewer contacts)?

- Yes
- No
- I do not know

9. If yes free text question: How were these groups of patients, who used the services of your practice in a significantly different way, characterized in terms of diagnoses, age group, new/previously known or other characteristics? And: Did these groups have more or less contacts?

Free text

10. Did certain groups of patients (both new and previously known) use the services of your practice in or after the 2nd HIP in a significantly different way (e.g. significantly more or fewer contacts)?

- Yes
- No
- I do not know

11. **If yes free text question: How were these groups of patients, who used the services of your practice in a significantly different way, characterized in terms of diagnoses, age group, new/previously known or other characteristics? And: Did these groups have more or less contacts?**

Free text

12. **Did certain groups of patients (both new and previously known) use the services of your practice in or after the 3rd HIP in a significantly different way (e.g. significantly more or fewer contacts)?**

- Yes
- No
- I do not know

13. **If yes free text question: How were these groups of patients, who used the services of your practice in a significantly different way, characterized in terms of diagnoses, age group, new/previously known or other characteristics? And: Did these groups have more or less contacts?**

Free text

14. **Did patients experience difficulties due to the pandemic-related adjustments in the outpatient and inpatient psychiatric-psychotherapeutic care system?**

- Yes
- No
- I do not know

15. **If yes: Which? (multiple answer possible)**

	1st HIP March/April 2020	2nd HIP November 2020 to February 2021	3rd HIP March to Mai 2021
Increased demand after the HIP			
Increased exacerbations/aggravations/relapses			
Increased suicide attempts			
Increased suicides			
Increased contact discontinuations/treatment discontinuations			
Other difficulties	Free text	Free text	Free text

16. Did you have to treat more patients in the three HIP due to a reduced offer of the responsible hospital (e.g. discharged prematurely or not admitted despite the need for treatment)?

- ○ Yes, in the 1st HIP (from March to May 2020)
- ○ Yes, in the 2nd HIP (from November 2020 to February 2021)
- ○ Yes, in the 3rd HIP (from March to May 2021)
- ○ No

c. Organization and economic situation:

17. Do you fear financial losses due to the pandemic? (Multiple answers possible)

- ○ Yes, because of reduced practice utilization
- ○ Yes, because of cost of protective measures
- ○ Yes, for other reasons: (free text)
- ○ No

18. Did you have the impression that you were sufficiently informed and supported by the Association of Statutory Health Insurance Physicians (Kassenärztliche Vereinigung)/the medical associations? (Multiple answers possible)

	Yes	No
Regarding hygiene standards		
Regarding protective equipment		
Regarding rapid tests		
Regarding telemedicine		
Regarding special billing codes		
Regarding other areas not sufficiently informed and supported	Free text	

d. SARS-CoV-2 infections and vaccinations in patients and employees:

19. Did you have contact with SARS-CoV-2 positive patients in your practice?

- ○ Yes
- ○ No

20. If Yes: Did you have to temporarily close your practice due to your own COVID-19 illness or to high absenteeism due to COVID-19 illness of the staff?

- ○ Yes
- ○ No

**21. Patients with severe mental illness can claim "high priority" COVID-19 vaccination.
For which risk groups do you see problems with them claiming the vaccination?**

Free text

**22. Patients with severe mental illness can claim "high priority" COVID-19 vaccination.
What structures and measures are needed to implement nationwide vaccination of these risk groups?**

Free text

e. Hospital admission behavior:

23. If selected psychiatry, psychosomatic medicine, psychiatry and neurology or neurology at 1: How has your admission behavior to inpatient treatment in psychiatry and psychosomatic medicine changed during the pandemic? (Multiple answers possible)

	Less patients admitted for inpatient treatment	Essentially remained the same	More patients admitted for inpatient treatment	Not assessable
1st HIP March/April 2020				
2nd HIP November 2020 to February 2021				
3rd HIP March to Mai 2021				

24. If "less" was selected at 23: What were the reasons for the reduced number of admissions? (Multiple answers possible)

- Less demand from patients due to fear of getting infected with SARS-CoV-2 in the hospital.
- Less demand from patients due to restrictions during inpatient treatment due to hygiene measures (reduced therapies, visiting opportunities, exits, etc.).
- Less demand from patients with lower disease burden (e.g., less severe exacerbations in HIP).
- Reduced supply of clinics (e.g., only admissions of emergencies and in cases of acute danger).
- Restraint on my part for fear of getting infected SARS-CoV-2 in the hospital.
- Restraint on my part due to the restrictions during inpatient treatment due to hygiene measures of the hospitals.
- Other reasons: Free text
- The reasons are unclear to me / I do not know.

f. Nursing homes and assisted living institutions:

25. If selected psychiatry, psychosomatic medicine, psychiatry and neurology or neurology at 1: Do you provide psychiatric care at nursing homes, homes for the elderly, or complementary care facilities such as group homes/residential projects for people with mental illness?

- Yes
- No If no, continue with g./question 31.

26. During the pandemic, how did the care you provided by nursing homes, homes for the elderly, or complementary care facilities such as housing groups/residential projects for people with mental illness change?

- More frequently medical visits
- Less frequently medical visits
- No change in medical visits

27. If "less frequently" is selected at 26: What were the reasons for less frequently medical visits on your part to old people's homes, nursing homes or complementary care facilities such as residential groups/residential projects for people with mental illness? (Multiple answers possible)

- The facility did not want any visits from me as a COVID-19 protective measure
- I visited as few facilities as possible as a COVID-19 protective measure
- Less demand from patients for decrease in mental illness or exacerbations
- Less demand from patients for fear of SARS-CoV-2 infection
- Less demand from patients due to COVID-19 outbreaks
- Less demand from patients because they were hospitalized for COVID-19
- Less demand from patients because they had died from COVID-19
- Fewer visits for other reasons: Free text
- The reasons are unclear to me / I do not know

28. Have there been changes in inpatient admissions for inpatient psychiatric/psychosomatic treatment from residential and complementary facilities? (Multiple answers possible)

- Yes, there was a change in the need for admissions
- No, essentially stayed the same
- I do not know

29. If yes: What changes have there been?

- In the first HIP, fewer patients were admitted for inpatient treatment
- In the second HIP, fewer patients were admitted for inpatient treatment
- In the third HIP, fewer patients were admitted for inpatient treatment
- More patients admitted for inpatient treatment in the first HIP
- More patients admitted for inpatient treatment in the second HIP
- More patients admitted for inpatient treatment in the third HIP

30. If for 29. "fewer": What were the reasons for the reduced number of admissions from residential and complementary institutions? (Multiple answers possible)

- Less demand due to fear of getting infected with SARS-CoV-2 in the hospital
- Less demand due to restrictions during inpatient treatment because of hospital hygiene measures (reduced therapies, visiting opportunities, exits, etc.)
- Less demand due to a lower disease burden (e.g., less severe exacerbations in the HIP)
- Less need for admission due to COVID-19 outbreaks or deaths in the homes/complementary facilities
- Fewer admissions because nursing home/complementary facility residents could be seen less frequently as part of COVID-19 protective measures
- Reduced clinic services (e.g., emergency, and acute threat admissions only)
- Restraint on my part for fear of infection with SARS-CoV-2 in the hospital
- Restraint on my part due to the restrictions during inpatient treatment due to hygiene measures of the hospitals
- The reasons are unclear to me / other reasons / I do not know

g. Telemedicine:

31. Do you use telemedicine services and if so, which ones?

	Telephone consultation/therapy	Video consultation/therapy	Selfhelp applications
Not used			
Already used before the pandemic			
Newly introduced during the pandemic			
Use also planned after the pandemic			
Introduction planned after the pandemic			

32. If at 31. "used" or "newly introduced": For which groups of patients were telemedicine services used in the pandemic and how were the experiences with them?

	F0: Dementias and deliriums	F1: Addictive disorders	F2: Psychoses	F3: Affective disorders	F4: Anxiety, obsessive-compulsive and stress disorders	F5: Eating disorders/ body-related disorders	F6: Personality disorders
Not used							
Good experiences							
Problematic experiences							

h. Post-COVID-Syndrome:

33. Have you treated patients whose mental illness you would classify as resulting from confirmed SARS-CoV-2 infection (post-COVID syndrome)?

- Yes **if yes: How many?** Free Text
- No

34. If "yes" at 33: What psychopathology occurred in the context of post-COVID syndromes?

- Disturbances of orientation, conception, concentration or mnestics
- Formal thought disorders other than rumination.
- Psychotic phenomena (delusions, sensory delusions/hallucinations/ego disorders)
- Rumination
- Depressed mood, decreased affective vibratory capacity, anhedonia
- Fatigue, decreased drive, lack of energy
- Anxiety
- Compulsive thoughts or actions.
- Psychomotor agitation, states of tension
- Increased irritability, aggressiveness
- Sleep disorders
- Intrusions or flashbacks
- Pain
- Weariness with life, suicidal thoughts
- Other: free text

35. If **"yes" at 33:** **What interventions did you use to treat post-COVID syndromes?**

- supportive conversations
- drug treatment with antidepressants
- drug treatment with highly potent neuroleptics
- drug treatment with sedative medications (e.g., benzodiazepines, Z-substances, low-potency neuroleptics)
- psychotherapeutic interventions (e.g., daily structure, activity schedules, cognitive restructuring, etc.)
- Mediation/application for rehabilitation treatments.
- Mediation/application for complementary treatments (e.g., outpatient occupational therapy, individual psychosocial support, etc.)
- Application for a retirement pension
- Other: Free text

i. Further and conclusion:

36. **What issues not asked about here arose in the pandemic and what good practice solutions have worked in your practice?**

Free text
