# Supplementary material for: Outpatient mental health care during high incidence phases of the COVID-19 pandemic in Germany – changes in utilization, challenges and post-COVID care
Source: Eur Arch Psychiatry Clin Neurosci. 2024 Sep 1;274(8):2025–35. doi: 10.1007/s00406-024-01886-w (PMC11579151; doi:10.1007/s00406-024-01886-w)
Supplement: Supplementary file 2 — (PDF 419 kb) [file 406_2024_1886_MOESM2_ESM.pdf]

Supplementary Information
Online Resource 2

Outpatient Mental Health Care During the First Three High Incidence Phases of the COVID-19 Pandemic in Germany - Results from the COVID Ψ Outpatient Survey

Mandy Fehr[1], Sabine Köhler[2,3], Christa Roth-Sackenheim[3,2], Katharina Geschke[1], Oliver Tüscher[1], Kristina Adorjan[4], Klaus Lieb[1], Lars P. Hölzel[1,5] and Hauke F. Wiegand[1]

[1]Department of Psychiatry and Psychotherapy, University Medical Center of the Johannes Gutenberg-University Mainz, Germany

[2]Berufsverband Deutscher Nervenärzte

[3]Berufsverband Deutscher Psychiater

[4] Department of Psychiatry and Psychotherapy, University Hospital, Ludwig Maximilians University Munich, Munich, Germany

[5]Oberberg Parkklinik Wiesbaden Schlangenbad, Schlangenbad, Germany

Keywords: COVID-19; pandemic; mental health care; outpatient care; psychiatry; telemedicine; Long COVID; COVID Vaccination

Corresponding author:

Hauke Felix Wiegand
Department of Psychiatry and Psychotherapy,
University Medical Center of the Johannes Gutenberg-University Mainz
Untere Zahlbacher Straße 8
55131 Mainz
Germany
+49 (0) 6131 177511
Haukefelix.wiegand@unimedizin-mainz.de

eTable 1

How were these groups of patients who made use of your practice's services characterized in terms of diagnoses, age group, new/previously known or other characteristics? And did these groups have more or fewer contacts? *(following the question: Did certain patient groups (both new and previously known) make significantly different use of your practice's services during or after the first high incidence phase of the pandemic (e.g. significantly more or fewer contacts)?*

Main- and subgroups	Examples
**1. Less contacts**	
F1	"Addiction patients have significantly fewer contacts"
F3	"fewer contacts, especially patients from the F3 and F4 diagnosis groups, more older patients"<br>" in some cases patients with e.g. anxiety disorders or depression no longer came "
F4	"Anxiety disorders came less frequently"<br>"Anxiety patients stayed at home more often, canceled the appointment"
Previously known patients vs. new patients	"All age groups, known patients, less frequent presentations and handling of necessary matters by telephone"<br>"Old, previously known patients had less contact"
Age of the patients (mainly older patients)	"Reduced contact by very old and multimorbid patients"<br>"Age group over approx. 65 years reduced contacts in the first wave"
Nursing homes and complementary facilities	"Less contact in retirement homes and residential facilities, limited to the absolute minimum and replaced by telephone calls and video consultations wherever possible"
**2. More contacts**	
F2	"People with schizophrenic disorders make significantly more use of the service"
F3	"Panic disorders and depressive disorders occurred more frequently"<br>"More contact with depressive patients"
F4	"Anxiety patients increased"<br>"More contacts, predominantly anxiety patients"
Previously known patients vs. new patients	"Many inquiries from new patients"<br>"Many other new patients, some of them patients who might never have presented to a psychiatric practice without the pandemic "
Use of telephone or video consultation	"More telephone contacts"<br>"Many patients wanted video or telephone contact"
Age of the patients (mainly younger patients)	"More contacts, predominantly younger people"<br>"Younger patients, mainly due to the multiple burdens of work and family during the pandemic. Very young adults with fears about the future; increased need"
**3. Diagnosis/change unclear**	"Anxiety disorders, depression, epilepsy"<br>"Old, multimorbid patients"
**5. Social isolation problem**	"Panic disorders and depressive disorders occurred more frequently, as well as stress reactions. The patients were very socially isolated"<br>"Students with problems due to online teaching, lack of social contacts, daily structure"
**4. Other**	"All patient groups"<br>"Contacts were more reliable, hardly any failures"

eTable 2

How were these groups of patients who made use of your practice's services characterized in terms of diagnoses, age group, new/previously known or other characteristics? And did these groups have more or fewer contacts? *(regarding the question: Did certain patient groups (both new and previously known) make significantly different use of your practice's services during or after the second high incidence phase of the pandemic (e.g. significantly more or fewer contacts)?*

Main- and subgroups	Examples
**1. Less contacts**	
F1	"Addiction patients have significantly fewer contacts"
F3	"Reduced contact from depressive and anxious patients"
F4	"Anxiety patients stayed at home more often, canceled appointments"<br>"Compulsive patients stayed at home"
Age of the patients (mainly older patients)	"Reduced contact by very old and polymorbid patients<br>polymorbid patients"<br>"Older people came less for fear of infection<br>of infection, telephone and video contacts could only<br>only compensate for this to a certain extent."
Previously known patients vs. new patients	"Old, previously known patients had less contact"
**2. More contacts**	
F2	"People with schizophrenic disorders significantly more utilization"
F3	"Significant increase in diagnoses of anxiety disorders, obsessive-compulsive disorders, depressive disorders"<br>"More contact, depression 40-60j"
F4	"Significant increase in diagnoses of anxiety disorders, obsessive-compulsive disorders, depressive disorders"<br>"More younger patients with anxiety and depressive disorders who are actually studying and working"
Use of telephone or video consultation	"More predominantly psychiatric video consultations - also with new patients"<br>"All patients made more use of the additional offered telephone consultation hours more (face-to-face contacts and telephone contacts)"
Age of the patients (mainly younger patients)	"More younger patients with anxiety and depressive disorders who are actually studying and working"<br>"More middle-aged groups affected due to strong effects of school closures, lack of leisure opportunities "
Social isolation	“Anxiety, older people with a lack of social contacts“
Parents (especially mothers)	"Mothers with multiple responsibilities working from home, children, loss of help from grandparents, social institutions"<br>"I have specialized in the treatment of mental illness in pregnancy and postpartum for over 20 years. Many mothers who I hadn't seen for a long time have returned with recurring or new complaints or massive symptoms of exhaustion"
Consequences of COVID-19	"Patients either had corona with subsequent complaints, illnesses delayed for fear of infection, corona-related "collateral damage" -> loneliness/grief reaction/overload/fear of the future"<br>"Long-Covid patients with significantly increased need for treatment"
**4. Other**	"Partly more, partly less"<br>"2nd and 3rd wave more questions of meaning and existence"

eTable 3

How were these groups of patients who made use of your practice's services characterized in terms of diagnoses, age group, new/previously known or other characteristics? And did these groups have more or fewer contacts? *(regarding the question: Did certain patient groups (both new and previously known) make significantly different use of your practice's services during or after the third high incidence phase of the pandemic (e.g. significantly more or fewer contacts)?*

Main and subgroups	Examples
**1. Less contacts**	
F4	"Anxiety disorders were less frequent"<br>"more anxious patients stayed at home and canceled appointments"
Age of the patients (mainly older patients)	"Reduced contact by very old and polymorbid patients"<br>"Age group over approx. 65 years reduced contacts in the first wave"
Previously known patients	"previously known patients have extended their appointments in order to have fewer contacts"
**2. More contacts**	
F3	"Significant increase in diagnoses of anxiety disorders, obsessive-compulsive disorders, depressive forms"<br>"More contact, depression 40-60j"
F4	"Significant increase in diagnoses of anxiety disorders, obsessive-compulsive disorders, depressive disorders"<br>"More younger patients with anxiety and depressive disorders who are actually studying and working"
Multiple loads	"More contact with very stressed patients due to the economic and social situation Multiple burdens of home office and homeschooling."
Previously known patients vs. new patients	"New patients have come in unchanged. More often from more distant districts"
Use of telephone or video consultation	"Demand for video consultations has increased."<br>"Overall, significantly more additional telephone contacts here too"
Age of the patients (mainly younger patients)	"Many young people asked for appointments; often very desperate."
Social isolation	"Both new and known patients; fewer contacts overall - sometimes very lonely"
Parents (especially mothers)	"Employees who work almost exclusively from home and who have few social contacts (mothers with multiple workloads working from home, children, loss of help from grandparents, social institutions)"
Consequences of COVID-19	"First-time patients with Long Covid syndromes"
Chronically ill people	"In 3rd wave severely chron. Sufferers exhausted, more crises, more contacts"
Catch-up effects	"There were now more patients for standard clarification who did not come in the first wave"
**4. Other**	"2nd and 3rd wave more questions of meaning and existence"

eTable 4

What other difficulties did patients experience? *(following the question: Did patients experience difficulties due to the pandemic-related adjustments to the outpatient and inpatient psychiatric-psychotherapeutic care system?)*

Main and subgroups	Examples
**Quitting social contacts**	"Breaking off social contacts"<br>"Loneliness"
**Limited career options**	"Lack of (professional) advancement, extension of illness phase and sick leave"
**Availability problems in the various sectors**	
Inpatient	"The psychiatric clinics were practically closed."<br>"Less frequent use of inpatient treatment"
Partial inpatient	"Partial inpatient services partly not possible although indicated."
Physician and psychotherapeutic outpatient	"Increase in the already pronounced shortage of psychotherapy services"
Rehabilitation	"Longer waiting times for ... especially psychosomatic rehab."
Complementary outpatient therapies	"Lack of presence self-help groups, lack of presence activities of social psychology services"
Psychosocial facilities/complementary facilities	" it was much more difficult to get good therapeutic offers, many services hardly have any offers anymore"
**Difficulties in treatment and in the doctor-patient relationship**	"In the second wave, I worked with masks, and patients with "early disorders" were unable to assess the conversation situation and my attitude (e.g. turned towards them) with confidence, which led to a loss of trust and security in the bond. There was more irritation in the relationship."
**Worsening symptoms and increased need for treatment**	"Worsening of symptoms."<br>"Chronification of anxiety disorders"
**Other**	"Increased demand for vaccination certificates"

eTable 5

Were there other issues in which you had the impression that you were not sufficiently informed and supported, and if so, which ones?

Super- and subgroups	Examples
**Proper information and support from the Association of Statutory Health Insurance Physicians**	„No"<br>„No"
COVID-19 vaccination	"The start of vaccinations was not well organized."<br>"The general public is poorly informed, misinformed or not informed at all about vaccinations in general, about vaccination against Covid, about normal vaccination reactions etc.. That was/is unhelpful and dangerous!"
Additional organizational effort	"Additional time expenditure due to special regulations with only short-term validity, frequent changes, complicated billing, e.g. of vaccination certificates, new billing codes"
Effort due to digital solutions	"Online options in particular very often fail due to technical problems and data protection requirements"
Lack of financial support	"The KVs have not expanded their budgets despite the correspondingly high demand, put doctors under pressure because of digitization although basic things are currently not working, e.g. the Internet speed leaves something to be desired, the KV connectors are partly unreliable"
Lack of support during implementation	"There was enough information, but we were left to implement it without support."
Communication deficits	"Due to the rapidly changing regulations, there were often difficulties as to which regulations were currently active."
**Difficulties at the beginning, good support over time**	"In the first wave, we felt alone, but as things progressed, I found that the KV provided very good and comprehensive information. From May onwards, I would answer the first three questions with yes"
Information needs of patients	"There was a great need for clarification, advice and information on the part of the patients. That's okay. That's one of our tasks (although it took a lot of time!). The public health services, politicians and doctors have provided too little factual information in the public media. For example, there was very poor communication about infection routes, measures ... communicated very poorly."
**Other**	"Restrictive: I am employed in the MVZ."

eTable 6

What problems not covered here occurred during the pandemic?

Main and subgroups	Examples
**Increased time requirements**	"Increased time requirement"<br>"Greatly increased need for advice"
**Implementation of hygiene measures: staff**	"Pay more and constant attention to hygiene, pay attention to the resilience of all employees. "
**Implementation of hygiene measures: facilities**	"The practice is not suitable for the situation due to limited space. We have tried to make the best of it"
**Conflicts about COVID-19 protection measures**	"For our staff, the enforcement of hygiene rules and the discussion about constantly changing regulations that we have not established are an even greater imposition than for us doctors."
**Increased staff and time expenditure**	"The "open consultation hours" were equalized in order to maintain distances, which worked well, but affected the working hours of the employees and therefore naturally caused increased costs. So when the pandemic ends, the system will change again."
**Reduction in social contacts and their consequences**	"Significant increase/recurrence of mental health problems even in previously stable patients as a result of social isolation/overload (home office/homeschooling), loss of social networks, structures due to the lockdown decisions"
**Anxiety symptoms among patients**	"Greatly increased need for counseling<br>Highly increased anxiety symptoms<br>Decompensation of unstable patients<br>Increased psychosocial stress due to political measures""
**Problems in trans-sectoral cooperation**	"Very poor communication on the part of the clinics regarding discharge planning, poor communication with LVR outpatient facilities "
**Increased administrative efforts**	"The simultaneous pushing through of already pointless digitization steps by the government with corresponding additional effort has made everything much more difficult and continues to do so (ePA etc.)"
**Financial losses**	"There were frequent cancellations in the 1st and 2nd wave, but no more in the 3rd."
**Reductions in services**	"Group therapy: I couldn't continue to use my rooms for this because the minimum distance couldn't be maintained. It took some time before I was able to rent suitable premises for group treatment."
**Vaccination of patients**	"Too little vaccine, too little compensation for vaccination"
**Other**	"None"

eTable 7

What good practice examples have proven effective in your practice?

Main and subgroups	Examples
**Telemedicine**	"I have good experience of initial psychiatric consultations using video consultations. This helped me a lot to get an impression of whether I was able to treat patients well and help them quickly despite limited resources."
**Equalization in waiting rooms**	“The "open consultation hours" were equalized in order to maintain distances, which worked well“
**Contacting patients**	“Initiating contact with patients who missed appointments.“
**Vaccination**	“We have already offered and vaccinated patients with severe mental illnesses.”
**Communication and involvement of employees**	"More discussions with staff, training, protection and remuneration of staff."
**Education**	"Education, reassurance, extended sick leave for patients, involvement of the environment"
**Treatment continuity**	"Offer of constant availability and support, also in personal contact, no retreat to the telephone on my part."
**Psychological well-being**	“Prioritizing personal psychological well-being. “
**Other**	“Good practice involved learning to constantly adapt and maximize flexibility.”
