# Supplementary material for: Outpatient mental health care during high incidence phases of the COVID-19 pandemic in Germany – changes in utilization, challenges and post-COVID care
Source: Eur Arch Psychiatry Clin Neurosci. 2024 Sep 1;274(8):2025–35. doi: 10.1007/s00406-024-01886-w (PMC11579151; doi:10.1007/s00406-024-01886-w)
Supplement: Supplementary file 3 — (PDF 168 kb) [file 406_2024_1886_MOESM3_ESM.pdf]

Online Resource 1

Distribution of participants across the federal states of Germany in which they work

Distribution of participants across the federal states of Germany	% of the participants n=105
Baden-Württemberg	14%
Bavaria	23%
Berlin - Brandenburg	17%
Hamburg	1%
Hesse	2%
Mecklenburg-West Pomerania	0%
Lower Saxony - Bremen	4%
North Rhine-Westphalia	10%
Saarland - Rhineland-Palatinate	6%
Saxony	2%
Saxony-Anhalt	0%
Schleswig-Holstein	1%
Thuringia	2%
Not specified	5%
No assignment to individual federal states possible	15%
